# 1865. Refractory Nontuberculous Mycobacterial Pulmonary Disease: Natural History and Burden of Illness in Patients from the US Bronchiectasis and NTM Research Registry (BRR)

**DOI:** 10.1093/ofid/ofad500.1693

**Published:** 2023-11-27

**Authors:** Timothy R Aksamit, Radmila Choate, David M Mannino, Amanda E Brunton, Ping Wang, Mariam Hassan

**Affiliations:** Mayo Clinic, Rochester, Minnesota; University of Kentucky College of Public Health, Lexington, Kentucky; University of Kentucky College of Public Health, Lexington, Kentucky; COPD Foundation, Miami, Florida; Insmed Incorporated, Bridgewater, New Jersey; Insmed Incorporated, Bridgewater, New Jersey

## Abstract

**Background:**

Nontuberculous mycobacterial pulmonary disease (NTMPD) is a chronic lung infection that is challenging to treat, especially refractory NTMPD. Data are limited on the natural history, risk profile, and burden of refractory NTMPD. The objective of this study was to evaluate the burden of illness (BOI) among patients with NTMPD who later develop refractory NTMPD vs those who do not.

**Methods:**

This was a retrospective analysis using BRR data. Patients treated for NTMPD were included and stratified as refractory (received oral clofazimine/bedaquiline or inhaled amikacin/ALIS, or remained sputum culture positive despite ≥ 6 months of treatment) or nonrefractory. Modified bronchiectasis severity index (mBSI) at baseline visit and BOI including comorbid conditions and surgeries at the treatment visit were compared by cross-sectional analysis between the two groups. Longitudinal analysis of BOI focused on exacerbations and hospitalizations in the refractory group at their pre-refractory visit and refractory visit (first visit meeting the refractory definition).

**Results:**

A total of 1,064 patients treated for NTMPD were included: 462 (43.4%) refractory and 602 (56.6%) nonrefractory. During the baseline period, the refractory group had more severe bronchiectasis (Table 1, mBSI, mean [SD], 7.3 [3.4] vs 6.7 [3.4]). At the time of treatment, bronchiectasis was more common in the refractory than nonrefractory (96.2% vs 89.7%) group, as were hemoptysis (26.6% vs 16.3%) and GERD (50.5% vs 39.9%). Except BMI, there were no statistically significant differences between the pre-refractory and nonrefractory treatment visits (Table 2). In the refractory group, exacerbations and hospitalizations remained high from the pre-refractory to refractory visit (45.6% vs 39.4% and 13.3% vs 12.8%, respectively). Oxygen and bronchial hygiene use increased from the pre-refractory to the refractory visit (5.2% vs 9.3% and 54.5% vs 61.7%, respectively) consistent with increased management of respiratory conditions when not responding to treatment (Table 2).

**Figure:**
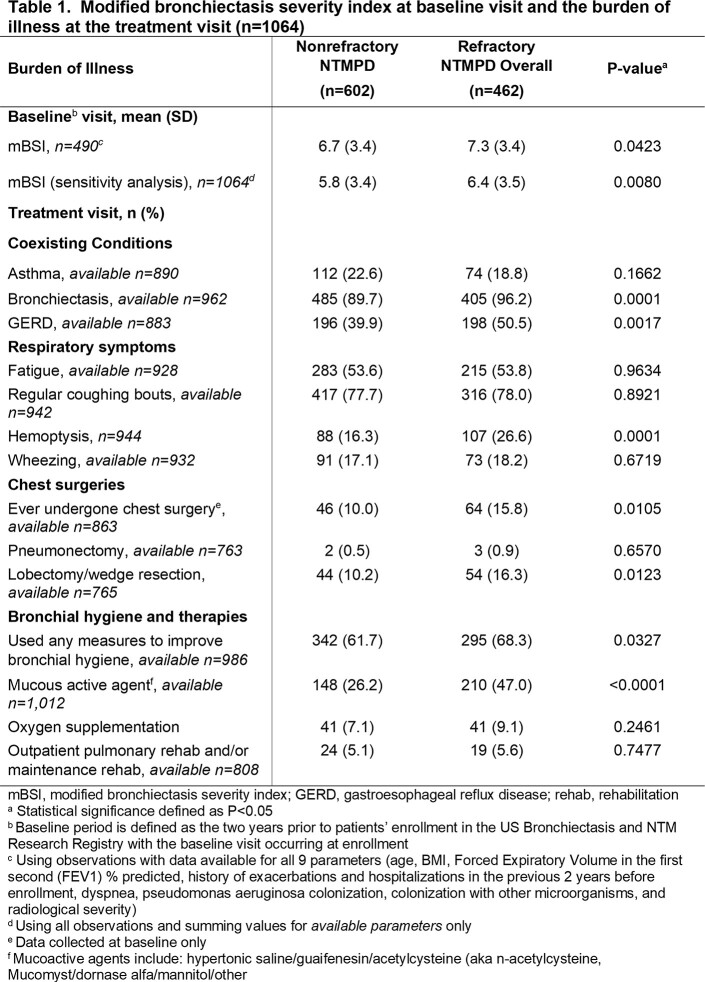

**Figure:**
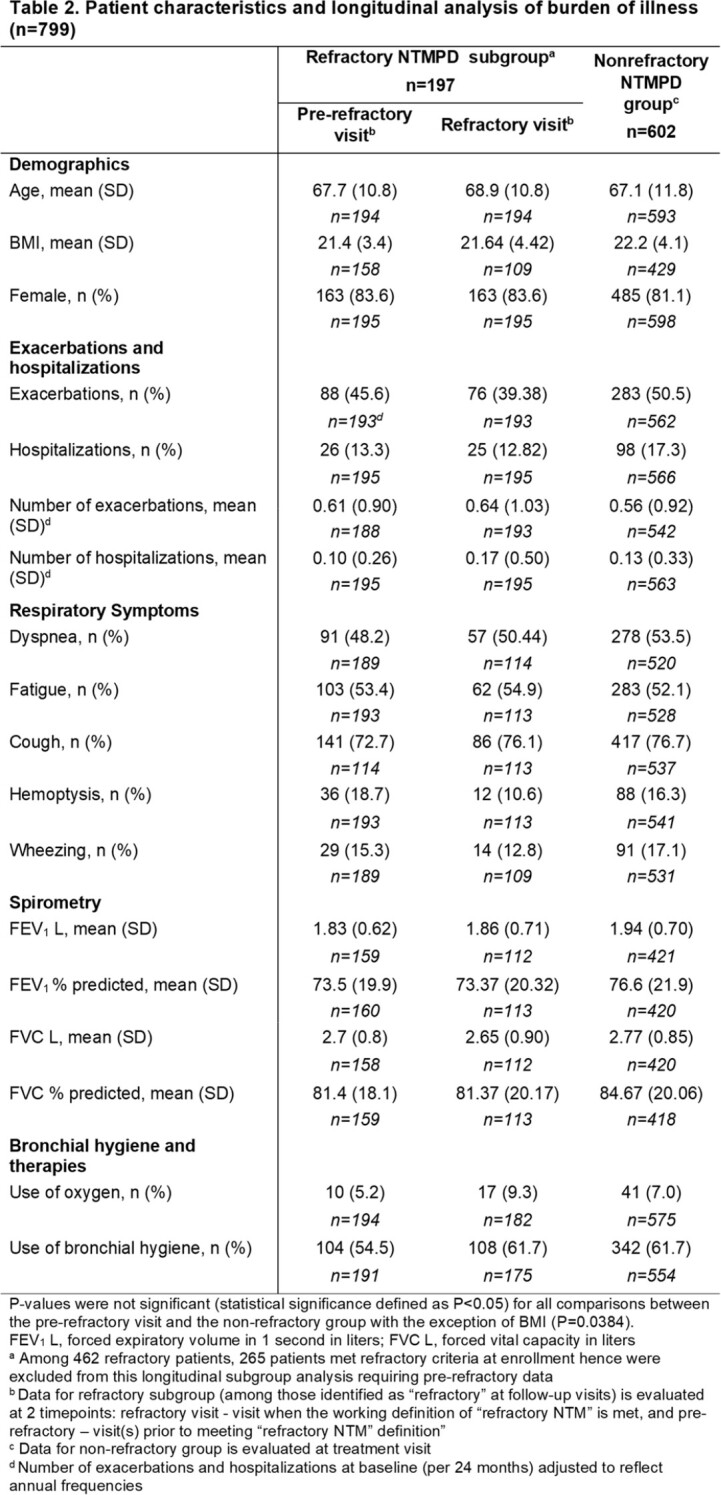

**Conclusion:**

Refractory NTMPD imposes a high disease burden on patients in the BRR. This high burden was present at pre-refractory visits and through the development of refractory status.

**Disclosures:**

**Timothy R. Aksamit, MD**, AstraZeneca: Advisor/Consultant|Baxter International, Hill-Rom: Advisor/Consultant|Insmed Incorporated: Advisor/Consultant|Johnson & Johnson: Advisor/Consultant|Redhill Biopharma: Advisor/Consultant|RespirTech: Advisor/Consultant|Spero Therapeutics: Advisor/Consultant|Zambon: Advisor/Consultant **David M. Mannino, MD**, AstraZeneca: Advisor/Consultant|GlaxoSmithKline: Advisor/Consultant|Up to Date: Advisor/Consultant **Ping Wang, PhD**, Insmed Incorporated: Salary|Insmed Incorporated: Stocks/Bonds **Mariam Hassan, PhD, B. Pharm**, Insmed Incorporated: Salary|Insmed Incorporated: Stocks/Bonds

